# Editorial: The cellular and molecular basis of nutrition and lifestyle in cancer prevention and progression

**DOI:** 10.3389/fonc.2025.1652368

**Published:** 2025-07-17

**Authors:** Adin Aggarwal, Maicon Roberto Kviecinski, Fabiana Ourique, Kenneth W. Yip

**Affiliations:** ^1^ Department of Cell and Systems Biology, University of Toronto, Toronto, ON,, Canada; ^2^ Department of Biochemistry, Biological Sciences Center, Federal University of Santa Catarina, Florianópolis, Santa Catarina, Brazil; ^3^ Department of Biochemistry, Institute of Biological Sciences, Federal University of Juiz de Fora, Juiz de Fora, Minas Gerais, Brazil

**Keywords:** nutrition, cancer, prevention, lifestyle, natural products, nutrients

## Overview

As the global cancer burden rises, nutrition and lifestyle remain underutilized approaches in both the prevention and treatment of cancer. The WHO currently estimates that 30-50% of cancer cases are preventable through nutrition and lifestyle changes (1). Furthermore, recent data shows that even post-diagnosis lifestyle modifications can significantly lower cancer-specific mortality (2). While some elements of a healthy lifestyle are well-established, such as eating fruits and vegetables and being physically active, the anticancer effects of specific micronutrients and phytochemicals remain less well understood. For instance, many such compounds are hypothesized to affect cancer outcomes through means such as modulating immune function, triggering cancer cell apoptosis, and preventing oxidative damage (3). In an effort to shed light on the possible clinical applications of these compounds, this Research Topic consists of articles that examine the mechanisms through which these bioactive compounds can reduce cancer risk and progression. This includes articles on selenium, *Aronia melanocarpa L.* (black chokeberry) peel extract, and a *Debronium huoshanense* polysaccharide, and their effects on different cancer subtypes. As well, this Research Topic includes an article that used a composite index of inflammatory and nutritional biomarkers to predict cancer outcomes, as it illustrates the powerful effect of lifestyle factors on cancer survivors’ long-term health.

## *Aronia melanocarpa L.* (black chokeberry) and breast carcinoma

*Aronia melanocarpa L.* is a plant that grows in eastern North America and produces black chokeberries which are rich in antioxidants. In particular, the abundance of anthocyanins in *Aronia melanocarpa L.* have driven claims regarding its anticancer and anti-inflammatory properties. Dvorska et al. sought to test these anticancer properties in animal and *in vitro* models of breast carcinoma. They conducted these experiments using a powdered extract from *Aronia melanocarpa* fruit peels. In one experiment, *aronia* extract was administered to a mammary adenocarcinoma mouse model, causing significant reductions in tumor volume and mitotic index activity compared to untreated mice. *In vitro* experiments with MCF-7 and MDA-MB-231 cell lines also revealed that *aronia* extract exerted anti-proliferative and pro-apoptotic properties by activating executioner caspases and increasing the Bax/Bcl-2 ratio. These experiments also found that aronia extract augmented the effectiveness of the chemotherapy drug epirubicin against these cell lines. Overall, these pro-apoptotic effects suggest that *Aronia melanocarpa L.* warrants further investigation for its use against breast cancers.

## *Dendrobium huoshanense* polysaccharide and colon cancer

*Dendrobium huoshanense* is a herb used in Chinese traditional medicine. Its main bioactive compound, the *Dendrobium huoshanense* polysaccharide (DHP), has been demonstrated to inhibit cancer cell proliferation. Yao et al. sought to elucidate the mechanisms underlying the anti-proliferative effects of DHP in colon cancer cells. Their paper demonstrated that DHP selectively inhibited HCT116 colon cancer cells, and was less effective against Caco-2 and IEC6 cell lines. DHP induced apoptosis in HCT116 cells by increasing intracellular reactive oxygen species and activating the mitochondrial apoptotic pathway as well as the Fas-FasL cell death pathway. By identifying the pathways responsible for the selective anticancer properties of DHP, this paper can help inform future targets for DHP in cancer therapy.

## Anticancer mechanisms of selenium in hepatocellular carcinoma

Selenium is an essential micronutrient best known for its role in redox metabolism. As a cofactor in the glutathione peroxidase and thioredoxin reductase classes of enzymes, it plays a key role in preventing oxidative stress, a driving cause of DNA damage and cancer development. Luo et al. conducted a comprehensive review of clinical, *in vivo*, and *in vitro* research on the protective functions of selenium against the development and progression of hepatocellular carcinoma (HCC). They detailed how the anti-inflammatory and antioxidant properties of selenium can help attenuate the risk of HCC caused by pro-inflammatory conditions such as obesity, diabetes mellitus, Non-Alcoholic Fatty Liver Disease, and Alcoholic Liver Disease. Beyond antioxidant functions, Luo et al. highlighted the role of selenium for enhancing immune function. For example, by helping suppress the replication of Hepatitis B virus (HBV), selenium can potentially reduce the risk of HBV-induced HCC. Luo et al. also reviewed the mechanisms by which selenium can reduce HCC progression. They detail how selenium can induce apoptosis through the Bcl-2/Cytochrome C/Caspase-mediated pathways, as well as prevent metastasis by decreasing expression of Vascular Endothelial Growth Factor and increasing Selenium Binding Protein 1 expression. Selenium may also help create an anti-tumor immune microenvironment via enhancing cytotoxic T cell function and driving M1 macrophage activation.

## Naples prognostic score and anemia in cancer survivors

The Naples Prognostic Score (NPS) accounts for the inflammatory and nutritional status of patients to predict cancer outcomes, as these factors can greatly shape tumor characteristics and immune responses (4). The NPS includes measures of serum albumin, total cholesterol, neutrophil to monocyte ratio, and monocyte to lymphocyte ratio. Wu et al. conducted a retrospective study of data from 3,143 cancer survivors in the National Health and Nutrition Examination Survey (NHANES) to determine if NPS scores were correlated with anemia in cancer survivors. They concluded that higher NPS scores were correlated with a higher probability of having anemia. This was also detrimental to the long-term health of cancer survivors, as those with anemia and NPS scores in the highest quartiles had a significantly increased risk of all-cause mortality. As the NPS score is largely derived from factors modifiable through nutrition and lifestyle changes, this paper provides a valuable perspective on their importance in long-term outcomes for cancer survivors.

## Conclusion

Overall, this Research Topic captures the immense diversity of ongoing research regarding the role of nutrition and lifestyle in cancer prevention and progression. This included original *in vitro* and *in vivo* research on phytochemicals against specific cancer subtypes, a review of the anticancer properties of selenium, and a look at potentially new prognostic tools for cancer patients. Collectively, these papers represent a growing interest in nutritional and lifestyle factors as effective, widely accessible approaches to preventing cancer and enhancing existing cancer therapies.
